# Metabolic and behavioral effects of mutant huntingtin deletion in Sim1 neurons in the BACHD mouse model of Huntington’s disease

**DOI:** 10.1038/srep28322

**Published:** 2016-06-23

**Authors:** Rana Soylu-Kucharz, Barbara Baldo, Åsa Petersén

**Affiliations:** 1 Translational Neuroendocrine Research Unit, Department of Experimental Medical Science, Lund University, BMC D11, SE-22184 Lund, Sweden

## Abstract

Hypothalamic pathology, metabolic dysfunction and psychiatric symptoms are part of Huntington disease (HD), which is caused by an expanded CAG repeat in the *huntingtin (HTT)* gene. Inactivation of mutant *HTT* selectively in the hypothalamus prevents the development of metabolic dysfunction and depressive-like behavior in the BACHD mouse model. The hypothalamic paraventricular nucleus (PVN) is implicated in metabolic and emotional control, therefore we here tested whether inactivation of mutant *HTT* in the PVN affects metabolic and psychiatric manifestations of HD in BACHD mice. BACHD mice were crossed with mice expressing Cre-recombinase under the Sim1 promoter (Sim1-Cre) to inactivate mutant *HTT* in Sim1 expressing cells, i.e. the PVN of the hypothalamus. We found that inactivation of mutant *HTT* in Sim1 cells had a sex-specific effect on both the metabolic and the psychiatric phenotype, as these phenotypes were no longer different in male BACHD/Sim1-Cre mice compared to wild-type littermates. We also found a reduced number of GnRH neurons specifically in the anterior hypothalamus and an increased testes weight in male BACHD mice compared to wild-type littermates. Taken together, expression of mutant HTT in Sim1 cells may play a role for the development of metabolic dysfunction and depressive-like behavior in male BACHD mice.

Huntington’s disease (HD) is a neurodegenerative disorder with formal diagnosis based on prominent motor symptoms and a positive gene test for an expansion of the CAG repeat in the huntingtin (*HTT*) gene^1^,^2^. The onset of HD occurs in midlife, is followed by 15–20 years of disease progression, and with no disease-modifying treatment available, inevitably leads to premature death^2^. Non-motor aspects of the disease such as psychiatric symptoms and metabolic dysfunction are common and often occur early in the disease process^3^. The development of these features cannot easily be explained by the pathology in the striatum that constitute the neurobiological substrate for the motor symptoms of the disease. Hypothalamic circuitries are important regulators of metabolism and emotion^4^,^5^,^6^. A number of neuroimaging studies using magnetic resonance imaging (MRI) and positron emission tomography (PET) of individuals with the mutant *HTT* gene indicated changes in the hypothalamus before the onset of motor symptoms of disease^7^,^8^. Neuropathological studies of postmortem hypothalami have indeed demonstrated selective effects on metabolism and emotion regulating neuropeptides with loss of orexin, oxytocin and vasopressin in clinical HD^9^,^10^,^11^. Interestingly, a recent report of a case who died before motor onset and without striatal pathology showed loss of oxytocin and vasopressin in the paraventricular nucleus (PVN) suggesting that the PVN could be affected very early on in HD^12^.

Given the important role of the hypothalamus for the regulation of metabolism and emotion in combination with emerging evidence of hypothalamic dysfunction in HD, it remains plausible that the expression of mutant HTT in the hypothalamus is important for the development of non-motor features^13^. Indeed, we recently demonstrated a causal relationship between the selective expression of mutant HTT in the hypothalamus and the development of a severe metabolic phenotype with insulin and leptin resistance in mice^14^. We also showed that a similar metabolic phenotype as well as depressive-like behavior could be prevented if mutant *HTT* was inactivated in the hypothalamus of BACHD mice^14^,^15^. This is a mouse model expressing full-length mutant HTT with LoxP sites enabling cell or region specific inactivation of *HTT* using Cre-recombinase (Cre)^16^. Yet, the critical neuronal population in the hypothalamus responsible for this effect remains to be identified. Oxytocin and vasopressin expressing neurons in the PVN have been implicated in both emotional and metabolic control^17^,^18^. We therefore hypothesized that expression of mutant HTT in PVN neurons would be critical for the development of the metabolic and psychiatric phenotypes in BACHD mice. In this study, we crossed BACHD with mice expressing Cre under a key transcription factor for PVN neurons (Sim1-Cre), which resulted in deletion of expression of mutant HTT in Sim1 cells in the PVN. As Sim1 is also discretely expressed in the posterior hypothalamic nuclei, lateral olfactory tract, the supraoptic nucleus, the lateral hypothalamus as well as medial and basomedial amygdala^19^, the contribution of mutant htt expression in these areas was also taken into account in the assessment of the behavioral, metabolic and cellular consequences of the genetic manipulation.

## Results and Discussion

### Deletion of mutant *HTT* in Sim1 expressing cells of the PVN of the hypothalamus in BACHD/Sim1-Cre mice

**Figure 1:**
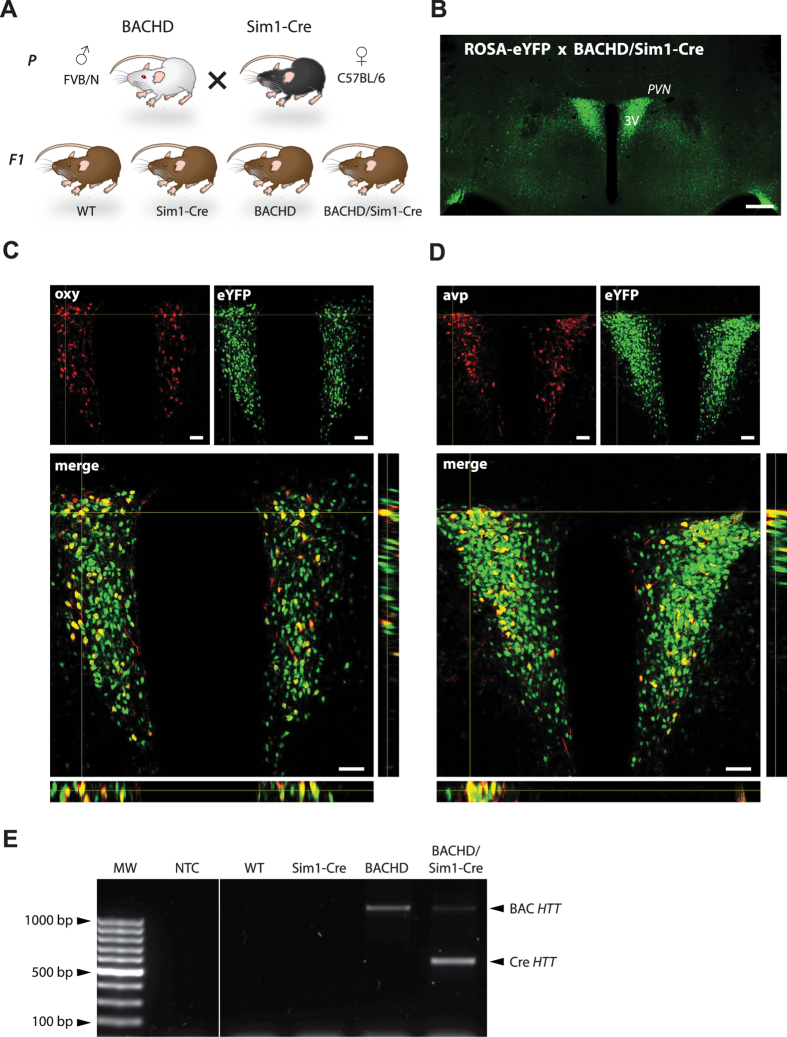
Deletion of mutant *HTT* in Sim1 expressing cells using a Cre/lox crossbreeding strategy. **(A)** Schematic illustration of the crossbreeding strategy and the offspring groups used in study. *P* = parents, *F1* = first generation offspring. **(B)** Confocal fluorescence imaging of endogenous expression of eYFP confirms the successful Cre-recombinase excision in the hypothalamus of ROSA-eYFP x BACHD/Sim1-Cre mice in the PVN, SON and in minor population of cells in the lateral hypothalamus. **(C**,**D)** Z-stack of fluorescence images showing co-localization of GFP (green) and oxytocin (oxy; red) or vasopressin (avp; red) in the PVN of ROSAxBACHD/Sim1-Cre mice. (**E)** Validation of mutant *HTT* excision in the PVN of BACHD/Sim1-Cre mice using PCR. *NTC* = non-template control. Scale bars represent 250 μm in B and 25 μm in C,D.

We crossed BACHD mice with Sim1-Cre mice in order to delete mutant *HTT* in Sim1 expressing neurons in the PVN of the hypothalamus (Fig. 1A). We first validated the specific expression and activity of Cre in the PVN of BACHD/Sim1-Cre mice by crossing them with a ROSA-eYFP mouse, which resulted eYFP expression in the PVN (Fig. 1B). We show that Cre expression was present and was functionally active in both oxytocin and vasopressin expressing neurons in the PVN by using immunofluorescence and confocal microscopy (Fig. 1C,D). We then confirmed that the successful Cre-mediated excised band of mutant *HTT* exon1 was present in the PVN of BACHD/Sim1-Cre mice (Fig. 1E). Finally, we examined the mRNA levels of Sim1 in the PVN of the four genotypes and found no differences in the expression levels (Table 1).

### The metabolic phenotype of male BACHD/Sim1-Cre mice is not different from male control mice

**Figure 2:**
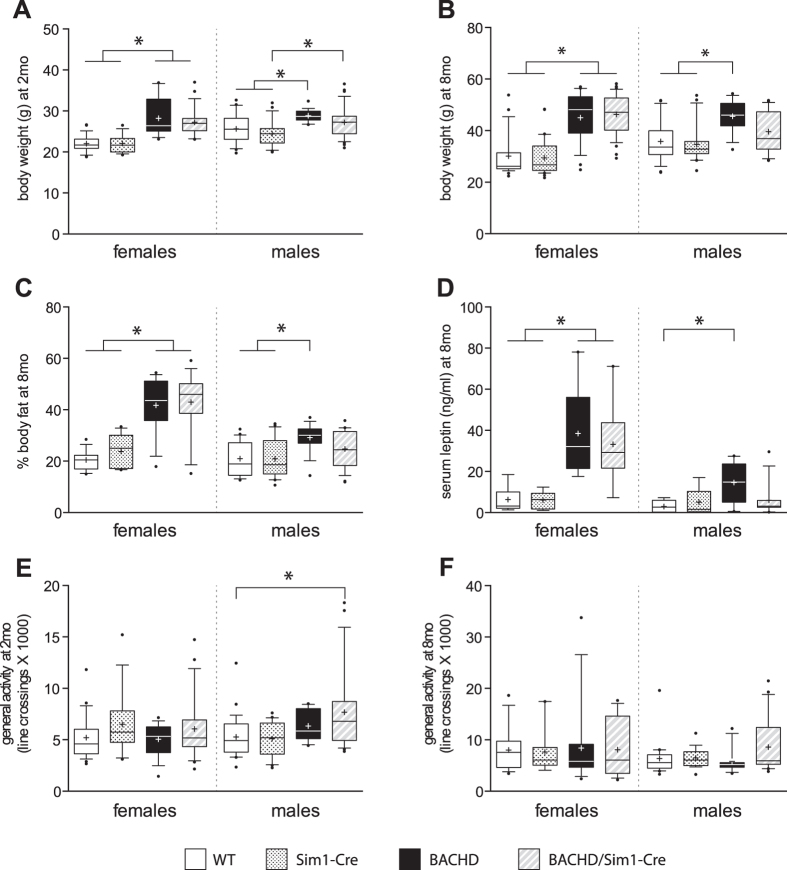
Sim1-Cre X BACHD metabolic phenotype. Body weight measurements at 2 months **(A)** (Females: n = 14–26/group, Kruskal-Wallis test followed by Dunn’s multiple comparison test; Males: n = 21–33/group, one-way ANOVA followed by Tukey’s posthoc test) and at 8 months **(B)** (Females: n = 28–36/group, males: n = 13–25/group, Kruskal-Wallis test followed by Dunn’s multiple comparison test). **(C)** DEXA scan analysis of body fat percentage at 8 months (Females: n = 13–19/group, Males: n = 16–25/group, Kruskal-Wallis test followed by Dunn’s multiple comparison test). **(D)** ELISA assessment of serum leptin levels. Both BACHD and BACHD/Sim1-Cre mice displayed elevated leptin levels compared to WT and Sim1-Cre controls in females. Only BACHD male mice showed increased serum leptin compared to WT control (Females: n = 8–9/group, one-way ANOVA followed by Tukey’s posthoc test; Males: n = 8–13/group, Kruskal-Wallis test followed by Dunn’s multiple comparison test). General motor activity assessment at 2 months (Females: n = 16–24/group, males: n = 14–26/group, Kruskal-Wallis test followed by Dunn’s multiple comparison test) **(E)** and at 8 months **(F)** (Females: n = 9–16/group, males: n = 11–21/group, Kruskal-Wallis test followed by Dunn’s multiple comparison test). Data are represented as box and whisker plots (25–75 percentile (boxes), 10–90 percentile (whiskers), median (horizontal line), mean (+), and outliers (dots)). *p < 0.05.

Next, we assessed the metabolic phenotype in our F1 cohort with both female and male mice of four genotypes. Both female and male BACHD mice displayed increased body weight, increased body fat and increased leptin levels compared to their control littermates (WT, Sim1-Cre) (Fig. 2A–D). Inactivation of mutant *HTT* in the hypothalamus of BACHD mice using an adeno-associated viral (AAV) vector expressing Cre under the general neuronal specific promoter synapsin has previously been shown to prevent the development of the metabolic phenotype^14^. The critical neuronal circuitry underlying this effect is not known. Here we show that deletion of mutant *HTT* in Sim1 neurons failed to prevent the obese phenotype in female BACHD mice. Notably, in the male group, the metabolic parameters of BACHD/Sim1-Cre mice were no longer different to the groups that do not express mutant *HTT*, i.e. WT and Sim1-Cre mice (Fig. 2A–D). When general activity was assessed, the only difference detected was between male BACHD/Sim1-Cre and WT mice at 2 month of age, when the BACHD/Sim1-Cre displayed a small increase in activity (Fig. 2E,F). Taken together, the data suggest that it is not the effect of mutant HTT expression in Sim1 expressing cells that underlies the metabolic phenotype in female BACHD mice whereas it cannot be excluded that it may play a role in male BACHD mice.

### The depressive-like phenotype of male BACHD/Sim1-Cre mice is similar to male control mice

**Figure 3:**
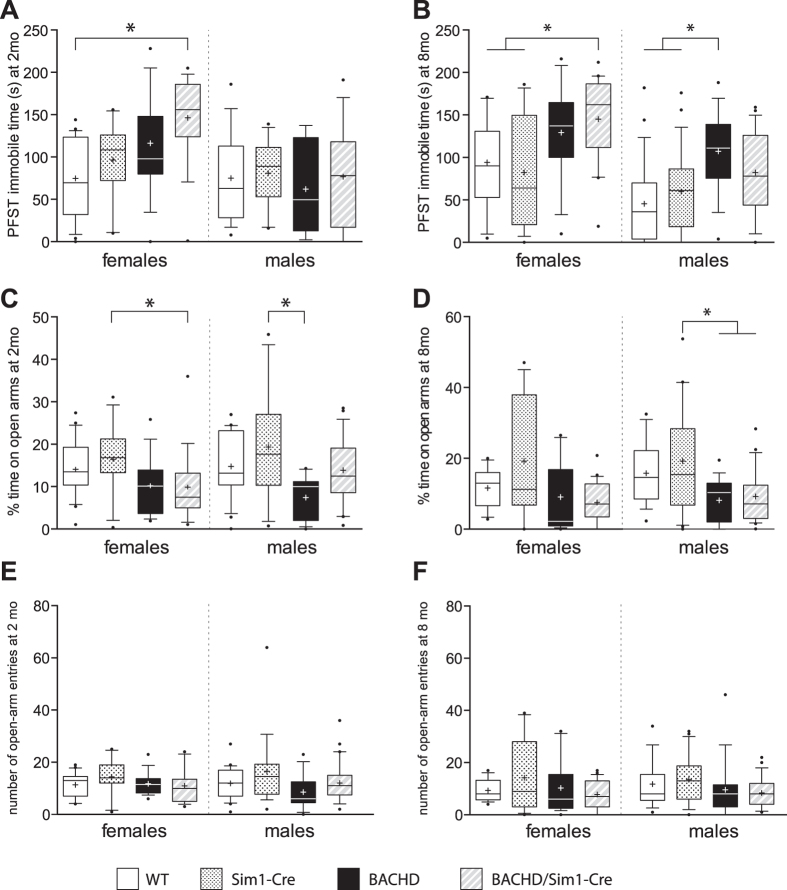
Evaluation of depressive and anxiety-like behaviors using the Porsolt forced swim test and the elevated plus maze test. **(A)** Female BACHD/Sim1-Cre mice displayed an increased immobility time spent in PFST compared to WT controls at 2 months (Females: n = 10–22/group, males: n = 6–18/group, one-way ANOVA followed by Tukey’s posthoc test). **(B)** An increased immobility time in PFST in BACHD/Sim1-Cre female and BACHD male mice compared to both WT and Sim1-Cre controls at 8 months (Females: n = 15–20/group, one-way ANOVA followed by Tukey’s posthoc test, Males: n = 17–25/group, Kruskal-Wallis test followed by Dunn’s multiple comparison test). **(C)** Female BACHD/Sim1-Cre and male BACHD mice displayed a decreased time spent on open arms at 2 months. (Females: n = 11–21/group, Kruskal-Wallis test followed by Dunn’s multiple comparison test; Males: n = 13–29/group, one-way ANOVA followed by Tukey’s posthoc test) **(D)** Analysis of the elevated plus maze test showed an decreased time spent on open arms in BACHD and BACHD/Sim1-Cre males compared to Sim1-Cre and no significant change in time spent on open arms between the genotypes in the female group at 8 months (Females: n = 15–22/group, Kruskal-Wallis test followed by Dunn’s multiple comparison test, Males: n = 17–23/group, one-way ANOVA followed by Tukey’s posthoc test). **(E)** Assessment of the number of open-arm entries showed no change between any of the groups at 2 months (Females: n = 11–21/group, one-way ANOVA followed by Tukey’s posthoc test; Males: n = 13–29/group, Kruskal-Wallis test followed by Dunn’s multiple comparison test) or **(F)** at 8 months of age (Females: n = 11–21/group, Kruskal-Wallis test followed by Dunn’s multiple comparison test; Males: n = 17–23/group, Kruskal-Wallis test followed by Dunn’s multiple comparison test). Data are represented as box and whisker plots (25–75 percentile (boxes), 10–90 percentile (whiskers), median (horizontal line), mean (+), and outliers (dots)). *p < 0.05.

BACHD mice have previously been shown to display a depressive-like behavior in the Porsolt forced swim test (PFST) and an anxiety-like behavior in the elevated-plus maze (EPM)^15^. Importantly, inactivation of mutant *HTT* in the hypothalamus of BACHD mice using AAV-vectors expressing Cre under the synapsin promoter prevented the development of depressive but no anxiety-like behavior in BACHD mice^15^. In the present study, female BACHD mice tended to spend more time immobile than their control littermates although this increase in immobility was only significant different between BACHD/Sim1-Cre and WT mice (Fig. 3A,B). Hence, this data argues against the hypothesis that expression of mutant HTT in Sim1 neurons would mediate the depressive–like behavior in female BACHD mice. The depressive-like phenotype as assessed using PFST was manifested first at 8 months of age in the male BACHD group in this study. Similarly as for the metabolic parameters, male BACHD/Sim1-Cre mice were neither statistically different to control groups nor to the BACHD group. Hence, a role of mutant HTT expression in Sim1 neurons for the development of depressive-like behavior in male BACHD mice cannot completely be ruled out.

The manifestation of the anxiety-like behavior previously demonstrated in BACHD mice was less clear in this cohort of mice. This may be due to effects of strain as we crossbred BACHD mice on a FVB/N strain with Sim1-Cre mice on a black 6 (B6) strain. In fact, in experiments, where diverse strains have been crossbreed, the penetrance of manifested phenotype in HD mice and the BACHD psychiatric symptoms may depend on combination of particular strains used in the breeding^20^,^21^,^22^,^23^,^24^. Nevertheless, the percentage time spent on open arms in the EPM, an indicator of anxiety-like behavior, was significantly reduced in both BACHD and BACHD/Sim1-Cre male mice compared to control littermates at 8 months of age (Fig. 3C,D). The number of entries into the open arms on the EPM was not different between the groups (Fig. 3E,F). Hence, the development of anxiety-like behavior is likely independent of mutant *HTT* expression in Sim1 neurons in male BACHD mice. There was also no apparent difference in anxiety-like behavior between female BACHD and BACHD/Sim1-Cre mice. This data supports previous experiments demonstrating that hypothalamic expression of mutant HTT is not involved in mediating anxiety-like behavior in BACHD mice.

### No changes in the numbers of oxytocin and vasopressin immunopositive neurons in the PVN of the hypothalamus in BACHD mice

Several transgenic animal models of HD recapitulate the loss of oxytocin and vasopressin in the PVN found in clinical HD^11^,^12^,^25^,^26^. Here, we first examined the mRNA expression levels of a number of genes expressed in the PVN. However, we did not find any differences in the levels of the PVN transcription factor Brn2 or neuropeptides such as oxytocin, vasopressin, GHRH, somatostatin (Table 1). Next, we assessed the number of oxytocin and vasopressin expressing neurons in the PVN of BACHD mice at 8 months of age. There was no difference in the number of oxytocin and vasopressin neurons between BACHD mice and wild-type littermates, and expression of Cre in these neurons did not affect their numbers (Supplementary Figure 1). Hence, the BACHD mice do not display the same alterations in the PVN as demonstrated in clinical HD.

### BACHD mice display reduced GnRH positive cells in the AHA and increased testes weight

**Figure 4:**
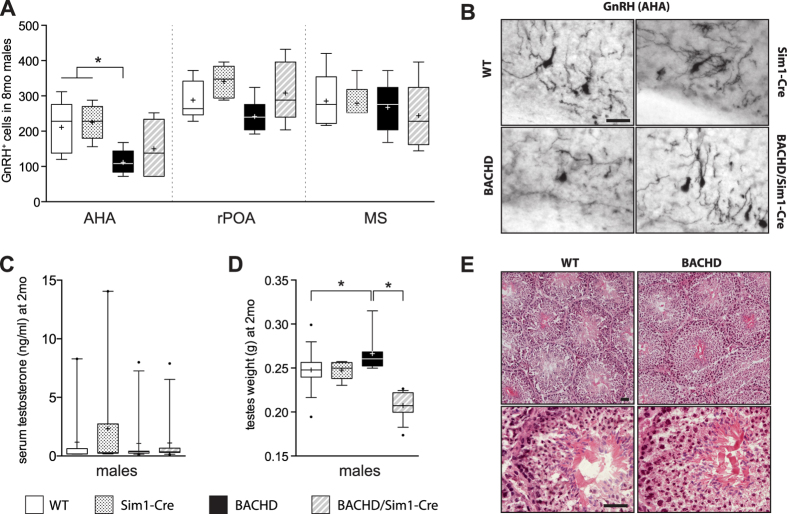
Assessment of the hypothalamic-pituitary-gonadal axis in male mice at 8 months. **(A)** Stereological quantification of GnRH positive cells in AHA (anterior hypothalamic area), rPOA (rostral preoptic area) and MS (medial septum) showed a significant reduction in BACHD males in AHA at 8 months. Number of GnRH positive cells in rPOA and MS were not different between genotypes (AHA and rPOA: n = 5–7/group, one-way ANOVA followed by Tukey’s posthoc test, MS: n = 5–7/group, Kruskal-Wallis test followed by Dunn’s multiple comparison test). **(B)** Representative photomicrographs showing GnRH positive cells in the AHA in WT, Sim1-Cre, BACHD and BACHD/Sim1-Cre mice. **(C)** No difference in levels of serum testosterone between different genotypes, as evaluated by ELISA (n = 9–11/group, Kruskal-Wallis test followed by Dunn’s multiple comparison test). **(D)** Increased testes weight in BACHD mice compared to WT, BACHD/Sim1-Cre and controls (n = 8–15/group, Kruskal-Wallis test followed by Dunn’s multiple comparison test). **(E)** Qualitative evaluation of hematoxylin and eosin stained testis sections of BACHD and WT mice showed normal morphology in BACHD mice at 8 months. Data are represented as box and whisker plots (25–75 percentile (boxes), 10–90 percentile (whiskers), median (horizontal line), mean (+), and outliers (dots)). Scale bars represent 25 μm. *p < 0.05.

Sex difference may be of importance for the studied phenotypes as inactivation of mutant *HTT* only had an effect in BACHD males. Therefore, we examined selected elements of the hypothalamic-pituitary-gonadal (HPG) axis in males, which regulates the release of sex hormones, i.e. testosterone. We assessed the numbers of GnRH positive cells in three distinct areas: anterior hypothalamic area (AHA), rostral preoptic area (rPOA) and medial septum (MS) in males of all four genotypes at 8 months of age. Our data show that BACHD males exhibit a significant reduction in GnRH neurons specifically in the AHA compared to the control animals (WT and Sim1-Cre) (Fig. 4A,B). Further analysis would be important to reveal whether the reduction in GnRH neurons reflects neuronal loss and/or reduced expression of the hormone itself.

Reduced release of GnRH leads to lower testosterone production in testes, which induces obesity with metabolic syndrome accompanied with emotional disturbances^27^. To examine if the loss of GnRH positive cells in AHA affected level of circulating testosterone in BACHD males, serum samples from all genotypes were analyzed with ELISA. Our data show that testosterone levels for all genotypes were comparable (Fig. 4C) and no differences between BACHD and WT males were found in testes morphology as assessed by hematoxylin and eosin staining (Fig. 4E). However, we found that BACHD group exhibited increased testes weight compared to WT and BACHD/Sim1-Cre mice (Fig. 4D). Parts of the HPG axis have been investigated previously in both clinical HD and in several animal models of the disease (Table 2). HD patients exhibit reduced testosterone levels in the late stages of disease, whereas GnRH tissue levels remain unaltered^28^,^29^. Furthermore, luteinizing hormone (LH) levels were reported to be decreased or unchanged while follicle-stimulating hormone (FSH) levels were found to be unaltered in clinical HD^28^,^30^,^31^. The testes of the HD patients become atrophied with reduced number of spermatids indicating deficit in spermatogenesis^32^. Testicular degeneration has been found also in the R6/2 and the YAC128 mouse models of HD with reduced levels of testosterone and decreased number of GnRH cells only in R6/2 mice^32^,^33^. LH and FSH levels have not yet been reported in HD mouse models. Hence, the data from the present study support previous findings of a perturbed HPG axis in HD although there is variability in the reported alterations in between models and in comparison to clinical material. This variability may be due to different background strain of the mice, different disease stages examined as well as different levels of HTT expression. Further studies are indeed warranted to determine the full characteristics and significance of HPG axis changes in HD.

Our results indicate that sex significantly affects the behavioral manifestation in BACHD mice. Sex-specific changes have been described also for other genetic HD animal models. In the R6/1 HD mice, only female mice show depressive-like behavior, which is linked with activation of the hypothalamic-pituitary-adrenal axis and dopaminergic dysfunction^34^,^35^,^36^. Male R6/1 mice display deficit in social interaction and reduced body weight, which is absent in female R6/1 mice^37^. The HdhQ111 HD mouse model shows increased anxiety-like behavior in the open field test, but only in males^38^. Thus, the results from this study further support that sex is another important factor that determines the manifestation and the extent of non-motor symptoms of HD pathology.

## Conclusions

In summary, inactivation of mutant *HTT* in Sim1 neurons of the hypothalamus and amygdala in BACHD mice affected the metabolic and depressive-like phenotype in a sex-specific manner. The manifestation of these phenotypes in BACHD/Sim1-Cre males was no longer different to control littermates, whereas deletion of mutant *HTT* in Sim1 neurons had no effect in BACHD/Sim1-Cre females. Our results also indicate a possible alteration in the HPG axis in BACHD male mice represented with a reduction of GnRH cells specifically in anterior hypothalamic area and increased testes weight, the latter was significantly reversed in BACHD/Sim1-Cre males.

## Materials and Methods

### Animals

BACHD mice from the FVB/N strain and B6.FVB(129 × 1)-Tg(Sim1-Cre)1Lowl/J (Sim1-Cre) were used in the study (the Jackson Laboratories). The BACHD mice express full-length mutant *HTT* and contain loxP sites flanking mutant *HTT* exon1, which enables inactivation of mutant *HTT* in specific cellular populations using Cre^16^. Sim1 (single-minded 1) is a transcription factor important for the development of the PVN but is also sparsely expressed in the other areas of the hypothalamus as well as the amygdala^19^,^39^. Sim1-Cre mice express Cre under the Sim1 promoter thereby inactivating mutant *HTT* expression in Sim1 expressing cells in BACHD/Sim1-Cre mice^19^. In this study, BACHD crossbred with Sim1-Cre mice resulted in total 4 different genotypes; WT, Sim1-Cre, BACHD and BACHD/Sim1-Cre. Both males and females of the F1 generation from crossbred of male BACHD and female Sim1-Cre mice were used in the study (Fig. 1A). To validate successful Cre activity in BACHD/Sim1-Cre mice, we crossed BACHD/Sim1-Cre mice with B6.129×1Gt(ROSA)26Sortm1(eYFP)Cos/J (C57BL/6 strain; Jackson laboratories). In the ROSA-eYFP mice, expression of Cre enzyme in the cells removes the floxed stop codon on the eYFP (enhanced yellow fluorescent protein) transgene and therefore activates eYFP expression^40^. The mice had free access to normal chow and were maintained at a 12 h light/dark cycle. The experimental procedures performed on mice were carried out in accordance with the approved guidelines in the ethical permit approved by the Lund University Animal Welfare and Ethics committee in the Lund-Malmö region (ethical permit numbers M20-11 and M65-13).

### PCR reaction

The deletion of mutant *HTT* exon 1 in the PVN of BACHD/Sim1-Cre was confirmed in genomic DNA from dissected PVN from brains of 2 months old mice. DNA extraction was performed using DNeasy Blood and Tissue kit according to manufacturer’s instructions (Qiagen). The PCR products were loaded on 1% agarose gel with 1% SYBR^®^ Safe DNA gel stain (Invitrogen). Specific primers sequences were designed for the detection of both excised and intact *HTT*, which were annealing on loxP sites of flanked *HTT* exon 1 Forward 5′-ATTCATTGCCCCGGTGCTGA-3′, Reverse 5′-AGCCCTCTTCCCTCTCAGACTAGAAGAGG-3′.

### Metabolic tests

For all animals used in study, body weight was measured at 2 and 8 months of age. The body fat content was determined at 8 months of age using dual energy x-ray absorptiometry (DEXA) scanning (Lunar PIXImus2, Lunar Corporation, Madison, WI, USA). Serum leptin and testosterone levels were assessed in blood collected from the heart left ventricle at 8 months of age. Collected blood was kept at room temperature for 30 minutes to clot, spun for 15 minutes at 2500 g and collected supernatant (serum) was stored at −80 °C in aliquots. Serum levels of leptin (Crystal Chem Inc, Catalog#90030) and testosterone (Demeditec, Cat.-No.: DEV9911) were determined with ELISA according to the manufacturer’s instructions.

### Behavioral tests

Mice were behaviorally tested both at 2 and 8 months of age using the following tests and in the following order: the open field test (OFT), the elevated plus maze (EPM) and the Porsolt forced swim test (PFST).

The OFT was used to assess general motor activity in mice at 2 and 8 months of age as previously described^41^. Animals were placed in the center of an activity box (40.6 cm × 40.6 cm) enclosed by transparent walls (38.1 cm). The activity boxes were connected to PAS-Open field test chambers (San Diego Instruments) and the general activity defined as the total number of beam crossings during the 60 minutes of recording.

The EPM was used to assess the anxiety-like phenotype in 2 and 8 months old mice as previously described^15^. Briefly, the platform consisted of cross-aligned four 6 cm wide and 30 cm long arms, with two arms of the same axis were enclosed by 30 cm high walls. The EPM platform was elevated 50 cm from its base. The EPM test relies on a basic conflict between exploring a novel area and aversion to an open space. A decrease in time spent in open area (non-walled arms of elevated plus maze) is considered as a sign of increase in anxiety-like behavior in mice^42^. The anxiety-like behavior (time spent on open arms) was monitored for 5 min in the EPM and registered with Ethovision 3.1 Software system (Noldus Information Technology).

The PFST was used to assess depressive-like behavior in mice at 2 and 8 months of age as previously described^15^. In brief, a glass transparent cylinder (height 18 cm, diameter 19 cm) was filled with water (~25 °C) up to 12 cm. Next, mice were placed in the center of the filled cylinder and the behavior was recorded for 6 min for every mice used in the study. The time of swimming activity is interweaved by periods when animals are passive and afloat. The passive behavior is considered as sign of helplessness and despair^43^. The first 2 minutes of recording was regarded as an acclimation period, whereas subsequent 4 minutes was evaluated for immobility as a sign of depressive-like behavior (Porsolt 1977).

### Immunohistochemistry

Sodium pentobarbital-anesthetized mice were perfused transcardially with physiological saline to rinse the vessels from blood and subsequently with pre-cooled 4% paraformaldehyde (PFA) for 10 min (at 10 ml/min). Next, brains were dissected and placed for 24 hours in 4% PFA solution at 4 °C for post-fixation then transferred to 25% sucrose solution at 4 °C for ~24–36 hours. The brains were sectioned on dry ice in the coronal plane (30 μm sections, six series per animal). Sectioned brains were stored at **−**20 °C in an antifreeze solution (30% glycerol, 30% ethylene glycol solution in phosphate buffer) until further processing.

To remove the antifreeze solution, the free floating brain sections were then rinsed 3 times for 10 minutes with 0.05 M tris (hydroxymethyl) aminomethane (Tris) -buffered saline (TBS). Following that, sections were left in TBS containing 3% H_2_O_2_ and 10% Methanol for a quenching reaction. Next, the sections were preincubated for 1 hour at the room temperature (RT) with blocking solutions containing 5% goat anti-rabbit and 0.25% triton-X in TBS (TBS-T) and then the brain sections were left in 5% serum in TBS-T containing primary antibody overnight at RT. The following primary antibodies and dilutions were used: anti-oxytocin (1:1000, anti-rabbit, Phoenix H-051-01), anti-vasopressin (1:10000, anti-rabbit, Chemicon), anti-GnRH (gonadotropin-releasing hormone: 1:3000, anti-rabbit, Abcam #ab5617), anti-GFP (1: 20000, anti-rabbit, Abcam #ab290). Prior to incubation with secondary antibodies (goat anti-rabbit, 1:200 dilution), the brain sections were rinsed 3 times for 10 minutes in TBS-T. Consequently, sections were washed with TBS and then incubated with an avidin-biotin-peroxidase complex solution (VECTASTAIN ABC Kit (Standard), Vector Laboratories Inc.) for 1h. 3,3′-diaminobenzidine (DAB) and 0.01% H_2_O_2_ was utilized to visualize the staining according to manufacturers instructions. Last, sections were mounted on chromatin-gelatin coated glass slides and secured with coverslips using Depex (Sigma-Aldrich).

### Confocal microscopy

For oxytocin (1:500 dilution, anti-rabbit, Phoenix) and vasopressin (1:5000, anti-rabbit, Chemicon) immunofluorescence staining, primary antibody incubations were performed overnight at RT. After rinsing 3 × 10 minutes with TBS-T, sections were incubated with secondary fluorescent antibody (DyLight 649 donkey anti-rabbit) for 1 hour at RT. Consequently, sections were mounted on gelatin coated slides and coverslipped with PVA-DABCO (Sigma-Aldrich). The imaging of endogenously expressed eYFP and Cy5 was performed with Nikon Eclipse Ti-E inverted laser scanning microscope (Nikon, Instruments Inc., Melville, NY) using 488 and 647 nm Sapphire laser lines. Apochromat 63x N.A. 1.40 oil immersion objective (Nikon) was used to collect Z-stacks of images. The data was acquired in single channel mode using Nikon EZ-C1 imaging software (v. 3.90) and processed as orthogonal projections with ImageJ (v.1.48u4; NIH).

### Gene expression analysis

The hypothalamic tissue was harvested immediately after decapitation and tissue dissection was performed ice. Samples were snap-frozen in liquid nitrogen and kept in −80 °C until further processing. RNA isolation was performed using the RNeasy Lipid Tissue Kit (Qiagen) and for 1 μg of RNA was used for the reverse transcriptase reaction (SuperScript III Reverse Transcriptase kit, Invitrogen) according the manufacturers instructions. SYBR Green-based detection (SYBR Green I Master, Roche) was utilized to measure gene expression changes and reaction was performed with a LightCycler 480 (Roche) in a two-step cycling protocol. To analyze the gene expression changes the comparative ΔΔCT method (ΔCt method) was employed. The two housekeeping genes used in the study were glyceraldehyde 3-phosphate dehydrogenase (GAPDH) and β-actin. Primer sequences are as follows: GAPDH: Forward: 5′-AACCTGCCAAGTATGATGA-3′, Reverse: 5′-GGAGTTGCTGTTGAAGTC-3′; β-actin Forward: 5′-GCTGTGCTATGTTGCTCTA-3′, Reverse: 5′-TCGTTGCCAATAGTGATGA-3′; Brn2: Forward: 5′-GTCACAGGAGATGCCATAGA-3′, Reverse: 5′-GCTTCGGACCTTACCTACTT-3′; Sim1: Forward: 5′-TCTTGGCTATCTGGTCTGA-3′, Reverse: 5′-CCTCTGCTTGTGAATGGA-3′; oxy: Forward: 5′-CCTACAGCGGATCTCAGA-3′, Reverse: 5′-CAGAGCCAGTAAGCCAAG-3′; Avp: Forward: 5′-ATGCTCAACACTACGCTCTC-3′, Reverse: 5′-GCAGTTCTGGAAGTAGCAGG-3′; Sst: Forward: 5′-TTCTGGAAGACATTCACATC-3′, Reverse: 5′-AGGAGTTAAGGAAGAGATATGG-3′; GHRH: Forward: 5′-ATCTTCACCACCAACTAC-3′, Reverse: 5′-ATGTCCTGGATCACTTTC-3′.

### Stereological analyses

Using the optical dissector method the unbiased stereological quantification principles were applied to estimate the numbers of cells positive for oxytocin and vasopressin in the PVN and for GnRH positive cells in the anterior hypothalamus (AHA), rostral preoptic area (rPOA), medial septum (MS)^44^. Stereological analyses were carried out with Nikon 80i microscope equipped with X–Y motorized stage (Märzhauser, Wetzlar) and a high precision linear encoder (Heidenhain, Traunreut). The region of interest was delineated under the 4X objective, whereas the counting was performed using a 60X NA 1.4 Plan-Apo oil objective with a random start systematic sampling routine (NewCast Module in VIS software; Visiopharm A/S, Horsholm). The position of the stage and the input from the digital camera were controlled by computer. The sampling interval was adjusted to count at least 100 cells for each hypothalamus to minimize the coefficient of error.

### Statistical analysis

All statistical analyses were performed using Prism 6 software (GraphPad). The data was initially tested with Kolmogorov–Smirnov test for normal distribution. Following that, the data was subjected to either the non-parametric Kruskal–Wallis followed by Dunn’s multiple-comparison test or the parametric one- or two-way analysis of variance (ANOVA) followed by Tukey’s multiple-comparison test. The statistical test results and the type of test used for each experiment are specified in detail in the supplemental statistical results section. Statistically significant differences were considered for p < 0.05. Data are presented as mean ± SEM.

## Additional Information

**How to cite this article**: Soylu-Kucharz, R. *et al*. Metabolic and behavioral effects of mutant huntingtin deletion in Sim1 neurons in the BACHD mouse model of Huntington’s disease. *Sci. Rep.* **6**, 28322; doi: 10.1038/srep28322 (2016).

## Supplementary Material

Supplementary Information

## Figures and Tables

**Table 1:** Hypothalamic gene expression analysis in male mice at 8 months.

Candidate Gene	Sim1-Cre (Percent of WT)	BACHD (Percent of WT)	BACHD/Sim1-Cre (Percent of WT)
Brn2	110 ± 5	113 ± 8	109 ± 6
Sim1	104 ± 7	110 ± 11	110 ± 8
Oxy	94 ± 9	87 ± 12	100 ± 6
Avp	77 ± 16	54 ± 6	68 ± 9
SST	85 ± 12	72 ± 4	71 ± 4
GHRH	76 ± 16	78 ± 3	74 ± 5

Data are expressed as mean ± SEM (n = 6–10/group, one-way ANOVA followed by Tukey’s posthoc test or Kruskal-Wallis test followed by Dunn’s multiple comparison test). Brn2: POU class 3 homeobox 2, Sim1: single minded 1, Oxy: oxytocin, AVP: vasopressin, SST: somatostatin, GHRH: growth hormone regulating hormone.

**Table 2:** Summary of hypothalamic-pituitary-gonadal axis changes in HD patients and in HD animal models.

	HD patients	R6/2	YAC128	BACHD
GnRH positive cells	N/A	↓	←→	↓
GnRH hormone	Males ←→Females ↑	N/A	N/A	N/A
Testes weight	N/A	↓	↓	↑
Testicular organization	Abnormal	Abnormal	Abnormal	Normal
Testosterone	Mild ←→Symptomatic ↓	↓	←→	←→
LH	↓ ←→	N/A	N/A	N/A
FSH	←→	N/A	N/A	N/A
References	28–32	33	32	

FSH: Follicle-stimulating hormone; GnRH: gonadotropin-releasing hormone; LH: Luteinizing hormone. N/A = not assessed.
