# Supplementary material for: Metabolic and behavioral effects of mutant huntingtin deletion in Sim1 neurons in the BACHD mouse model of Huntington’s disease
Source: Sci Rep. 2016 Jun 23;6:28322. doi: 10.1038/srep28322 (PMC4917832; doi:10.1038/srep28322)
Supplement: Supplementary Information [file srep28322-s1.pdf]

Metabolic and behavioral effects of mutant huntingtin deletion in Sim1 neurons in the BACHD mouse model of Huntington's disease

Rana Soylu-Kucharz[1*], Barbara Baldo[1] and Åsa Petersén[1]

1. Translational Neuroendocrine Research Unit, Department of Experimental Medical Science, Lund University, BMC D11, SE-22184 Lund, Sweden

Supplementary Figure 1: Numbers of oxytocin and vasopressin positive neurons in the PVN.

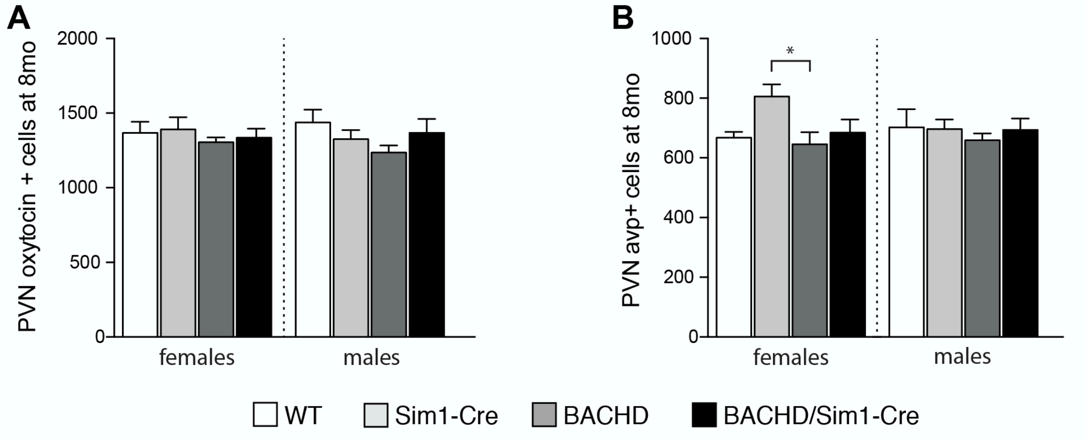

Supplemental Figure 1: Numbers of oxytocin and vasopressin positive neurons in the PVN. (A) Stereological assessment of PVN oxytocin positive cells showed no difference between any of the groups or sex (Females: n=4/group, Males: n=4-7/group, Kruskal-Wallis test followed by Dunn's multiple comparison test). **(B)** Sim1-Cre females showed significantly increased number of vasopressin positive cells in the PVN compared to BACHD females (Females: n=4/group, Males: n=4-7/group, Kruskal-Wallis test followed by Dunn's multiple comparison test). Data are expressed as mean±SEM. *$p<0.05$

Supplementary Statistical Results

The data was tested for normal distribution using a Kolmogorov–Smirnov test. $p<0.05$ was considered statistically significant and statistical analyses were performed using Prism 6 software (GraphPad). M: mean, SD: standard deviation.

Figure 2: Sim1-Cre X BACHD metabolic phenotype.

A. Females body weight measurement at 2 months

Kruskal-Wallis test: p< 0.0001
WT: M=22.04, SD=1.968, n=26
Sim1-Cre: M=22.07, SD=2.099, n=14
BACHD: M=28.22, SD=4.6, n=15
BACHD/Sim1-Cre: M=27.23, SD=3.319, n=22

Dunn's multiple comparisons test:
WT vs. Sim1-Cre: p> 0.9999
WT vs. BACHD: p< 0.0001
WT vs. BACHD/Sim1-Cre: p< 0.0001
Sim1-Cre vs. BACHD: p=0.0002
Sim1-Cre vs. BACHD/Sim1-Cre: p< 0.0001
BACHD vs. BACHD/Sim1-Cre: p> 0.9999

B. Males body weight measurement at 2 months

One-way ANOVA, F (3, 93) = 7.005. p=0.0003
WT: M=25.63, SD=3.491, n=26
Sim1-Cre: M=24.43, SD=3.05, n=21
BACHD: M=28.86, SD=1.451, n=27
BACHD/Sim1-Cre: M=27.22, SD=3.778, n=33

Tukey's multiple comparisons test:
WT vs. Sim1-Cre: p=0.5892
WT vs. BACHD: p=0.0103
WT vs. BACHD/Sim1-Cre: p=0.2512
Sim1-Cre vs. BACHD: p=0.0004
Sim1-Cre vs. BACHD/Sim1-Cre: p=0.0144
BACHD vs. BACHD/Sim1-Cre: p=0.3308

B. Females body weight measurement at 8 months

Kruskal-Wallis test: p< 0.0001
WT: M=30.14, SD=8.39, n=28
Sim1-Cre: M=29.45, SD=6.89, n=30
BACHD: M=45, SD=9.59, n=29
BACHD/Sim1-Cre: M=46.27, SD=7.62, n=36

Dunn's multiple comparisons test:
WT vs. Sim1-Cre: p> 0.9999
WT vs. BACHD: p=0.0001
WT vs. BACHD/Sim1-Cre: p=< 0.0001
Sim1-Cre vs. BACHD: p< 0.0001
Sim1-Cre vs. BACHD/Sim1-Cre: p< 0.0001
BACHD vs. BACHD/Sim1-Cre: p> 0.9999

B. Males body weight measurement at 8 months

Kruskal-Wallis test: p=0.0007
WT: M=35.8, SD=7.836, n=24
Sim1-Cre: M=34.65, SD=7.037, n=20
BACHD: M=45.57, SD=5.951, n=13
BACHD/Sim1-Cre: M=39.58, SD=7.883, n=25

Dunn's multiple comparisons test:
WT vs. Sim1-Cre: p> 0.9999
WT vs. BACHD: p=0.0033
WT vs. BACHD/Sim1-Cre: p=0.5416
Sim1-Cre vs. BACHD: p=0.0013
Sim1-Cre vs. BACHD/Sim1-Cre: p=0.2438
BACHD vs. BACHD/Sim1-Cre: p=0.2351

C. Females DEXA scan measurement at 8 months.

Kruskal-Wallis test: p< 0.0001
WT: M=20.46, SD=3.77, n=19
Sim1-Cre: M=23.81, SD=6.595, n=13
BACHD: M=41.78, SD=10.79, n=14
BACHD/Sim1-Cre: M=42.99, SD=11.41, n=18

Dunn's multiple comparisons test:
WT vs. Sim1-Cre: p> 0.9999
WT vs. BACHD: p=0.0001
WT vs. BACHD/Sim1-Cre: p< 0.0001
Sim1-Cre vs. BACHD: p=0.0051
Sim1-Cre vs. BACHD/Sim1-Cre: p=0.0016
BACHD vs. BACHD/Sim1-Cre: p> 0.9999

C. Males DEXA scan measurement at 8 months.

Kruskal-Wallis test: $p=0.0017$
WT: M=20.93, SD=7.351, n=24
Sim1-Cre: M=20.94, SD=6.663, n=24
BACHD: M=29.15, SD=5.462, n=16
BACHD/Sim1-Cre: M=24.74, SD=7.303, n=25

Dunn's multiple comparisons test:
WT vs. Sim1-Cre: $p> 0.9999$
WT vs. BACHD: $p=0.0041$
WT vs. BACHD/Sim1-Cre: $p=0.3013$
Sim1-Cre vs. BACHD: $p=0.007$
Sim1-Cre vs. BACHD/Sim1-Cre: $p=0.4445$
BACHD vs. BACHD/Sim1-Cre: $p=0.5597$

D. Females serum leptin measurement at 8 months.

One-way ANOVA, $F(3, 30) = 11.60$, $p< 0.0001$
WT: M=6.306, SD=6.047, n=8
Sim1-Cre: M=6.05, SD=4.086, n=8
BACHD: M=38.46, SD=20.72, n=9
BACHD/Sim1-Cre: M=33.12, SD=18.4, n=9

Tukey's multiple comparisons test:
WT vs. Sim1-Cre: $p> 0.9999$
WT vs. BACHD: $p=0.0005$
WT vs. BACHD/Sim1-Cre: $p=0.004$
Sim1-Cre vs. BACHD: $p=0.0005$
Sim1-Cre vs. BACHD/Sim1-Cre: $p=0.0037$
BACHD vs. BACHD/Sim1-Cre: $p=0.8679$

D. Males serum leptin measurement at 8 months.

Kruskal-Wallis test: $p=0.0259$
WT: M=2.859, SD=2.835, n=8
Sim1-Cre: M=5.003, SD=6.3, n=8
BACHD: M=14.61, SD=9.748, n=11
BACHD/Sim1-Cre: M=5.962, SD=7.724, n=13

Dunn's multiple comparisons test:
WT vs. Sim1-Cre: $p> 0.9999$
WT vs. BACHD: $p=0.0317$
WT vs. BACHD/Sim1-Cre: $p> 0.9999$
Sim1-Cre vs. BACHD: $p=0.1427$
Sim1-Cre vs. BACHD/Sim1-Cre: $p=> 0.9999$
BACHD vs. BACHD/Sim1-Cre: $p=0.364$

E. Females locomotor activity measurement at 2 months.

Kruskal-Wallis test: p=0.3656
WT: M=5205, SD=2147, n=24
Sim1-Cre: M=6496, SD=3105, n=16
BACHD: M=5047, SD=1594, n=16
BACHD/Sim1-Cre: M=6059, SD=3045, n=21

Dunn's multiple comparisons test:
WT vs. Sim1-Cre: p=0.5882
WT vs. BACHD: p> 0.9999
WT vs. BACHD/Sim1-Cre: p> 0.9999
Sim1-Cre vs. BACHD: p> 0.9999
Sim1-Cre vs. BACHD/Sim1-Cre: p> 0.9999
BACHD vs. BACHD/Sim1-Cre: p> 0.9999

E. Males locomotor activity measurement at 2 months.

Kruskal-Wallis test: p=0.0097
WT: M=5281, SD=2039, n=26
Sim1-Cre: M=5109, SD=1654, n=22
BACHD: M=6346, SD=1505, n=14
BACHD/Sim1-Cre: M=7678, SD=3947, n=23

Dunn's multiple comparisons test:
WT vs. Sim1-Cre: p> 0.9999
WT vs. BACHD: p=0.2212
WT vs. BACHD/Sim1-Cre: p=0.0285
Sim1-Cre vs. BACHD: p=0.4101
Sim1-Cre vs. BACHD/Sim1-Cre: p=0.0789
BACHD vs. BACHD/Sim1-Cre: p> 0.9999

F. Females locomotor activity measurement at 8 months.

Kruskal-Wallis test: p=0.8875
WT: M=8022, SD=4235, n=16
Sim1-Cre: M=7654, SD=4050, n=9
BACHD: M=8384, SD=8284, n=12
BACHD/Sim1-Cre: M=8089, SD=5602, n=15

Dunn's multiple comparisons test:
WT vs. Sim1-Cre: p> 0.9999
WT vs. BACHD: p> 0.9999

WT vs. BACHD/Sim1-Cre: p> 0.9999
Sim1-Cre vs. BACHD: p> 0.9999
Sim1-Cre vs. BACHD/Sim1-Cre: p> 0.9999
BACHD vs. BACHD/Sim1-Cre: p> 0.9999

F. Males locomotor activity measurement at 8 months.

Kruskal-Wallis test: p=0.2485
WT: M=6350, SD=3337, n=21
Sim1-Cre: M=6496, SD=1913, n=19
BACHD: M=5814, SD=2332, n=11
BACHD/Sim1-Cre: M=8576, SD=5205, n=20

Dunn's multiple comparisons test:
WT vs. Sim1-Cre: p> 0.9999
WT vs. BACHD: p> 0.9999
WT vs. BACHD/Sim1-Cre: p> 0.9999
Sim1-Cre vs. BACHD: p=0.802
Sim1-Cre vs. BACHD/Sim1-Cre: p> 0.9999
BACHD vs. BACHD/Sim1-Cre: p=0.463

Figure 3: Evaluation of depressive and anxiety-like behavior using Porsolt forced swim and elevated plus maze tests.

A. Females time spent immobile (s) in FST at 2 months.

One-way ANOVA, F (3, 60) = 6.695, p =0.0006
WT: M=74.73, SD=46.7, n=22
Sim1-Cre: M=96.4, SD=47.3, n=10
BACHD: M=116.3, SD=58.13, n=15
BACHD/Sim1-Cre: M=146.4, SD=50.84, n=17

Tukey's multiple comparisons test:
WT vs. Sim1-Cre: p=0.679
WT vs. BACHD: p=0.079
WT vs. BACHD/Sim1-Cre: p=0.0003
Sim1-Cre vs. BACHD: p=0.7714
Sim1-Cre vs. BACHD/Sim1-Cre: p=0.0747
BACHD vs. BACHD/Sim1-Cre: p=0.3469

A. Males time spent immobile (s) in FST at 2 months

One-way ANOVA, F (3, 51) = 0.1896, p =0.09030
WT: M=75.06, SD=52.24, n=18

Sim1-Cre: M=80.93, SD=40.12, n=14
BACHD: M=62, SD=55.15, n=6
BACHD/Sim1-Cre: M=76.76, SD=58.71, n=17

Tukey's multiple comparisons test:
WT vs. Sim1-Cre: p=0.9888
WT vs. BACHD: p=0.9506
WT vs. BACHD/Sim1-Cre: p=0.9997
Sim1-Cre vs. BACHD: p=0.8777
Sim1-Cre vs. BACHD/Sim1-Cre: p=0.9961
BACHD vs. BACHD/Sim1-Cre: p=0.9321

B. Females time spent immobile (s) in FST at 8 months.

One-way ANOVA, F (3, 63) = 4,984, p =0.0036
WT: M=94.06, SD=52.49, n=16
Sim1-Cre: M=82.31, SD=64.75, n=16
BACHD: M=129.2, SD=53.49, n=15
BACHD/Sim1-Cre: M=145.2, SD=49.63, n=20

Tukey's multiple comparisons test:
WT vs. Sim1-Cre: p=0.9306
WT vs. BACHD: p=0.2947
WT vs. BACHD/Sim1-Cre: p=0.0362
Sim1-Cre vs. BACHD: p=0.0937
Sim1-Cre vs. BACHD/Sim1-Cre: p=0.0062
BACHD vs. BACHD/Sim1-Cre: p=0.83

B. Males time spent immobile (s) in FST at 8 months.

Kruskal-Wallis test: p=0.0007
WT: M=45.36, SD=49.97, n=25
Sim1-Cre: M=59.88, SD=48.44, n=25
BACHD: M=107.2, SD=46.15, n=17
BACHD/Sim1-Cre: M=82.17, SD=48.7, n=23

Dunn's multiple comparisons test:
WT vs. Sim1-Cre: p> 0.9999
WT vs. BACHD: p=0.0007
WT vs. BACHD/Sim1-Cre: p=0.0536
Sim1-Cre vs. BACHD: p=0.0312
Sim1-Cre vs. BACHD/Sim1-Cre: p=0.837
BACHD vs. BACHD/Sim1-Cre: p=0.9463

C. Females percent time spent at open arms in EPM at 2 months.

Kruskal-Wallis test: p=0.0007
WT: M=14.13, SD=6.498, n=21
Sim1-Cre: M=16.49, SD=7.858, n=11
BACHD: M=10.26, SD=6.68, n=16
BACHD/Sim1-Cre: M=9.916, SD=8.085, n=18

Dunn's multiple comparisons test:
WT vs. Sim1-Cre: p> 0.9999
WT vs. BACHD: p=0.5226
WT vs. BACHD/Sim1-Cre: p=0.1106
Sim1-Cre vs. BACHD: p=0.1516
Sim1-Cre vs. BACHD/Sim1-Cre: p=0.0322
BACHD vs. BACHD/Sim1-Cre: p> 0.9999

C. Males percent time spent at open arms in EPM at 2 months.

One-way ANOVA, F (3, 79) = 4.512, p =0.0057
WT: M=14.76, SD=7.725, n=23
Sim1-Cre: M=19.34, SD=13.3, n=18
BACHD: M=7.409, SD=5.112, n=13
BACHD/Sim1-Cre: M=13.86, SD=7.785, n=29

Tukey's multiple comparisons test:
WT vs. Sim1-Cre: p=0.3709
WT vs. BACHD: p=0.092
WT vs. BACHD/Sim1-Cre: p=0.9838
Sim1-Cre vs. BACHD: p=0.0025
Sim1-Cre vs. BACHD/Sim1-Cre: p=0.1824
BACHD vs. BACHD/Sim1-Cre: p=0.1439

D. Females percent time spent at open arms in EPM at 8 months.

Kruskal-Wallis test: p=0.0961
WT: M=11.63, SD=5.562, n=18
Sim1-Cre: M=19.21, SD=17.41, n=15
BACHD: M=9.075, SD=9.707, n=17
BACHD/Sim1-Cre: M=7.521, SD=5.599, n=22

Dunn's multiple comparisons test:
WT vs. Sim1-Cre: p> 0.9999
WT vs. BACHD: p=0.8498
WT vs. BACHD/Sim1-Cre: p=0.3154
Sim1-Cre vs. BACHD: p=0.7247
Sim1-Cre vs. BACHD/Sim1-Cre: p=0.2751
BACHD vs. BACHD/Sim1-Cre: p> 0.9999

D. Males percent time spent at open arms in EPM at 8 months.

One-way ANOVA, F (3, 73) = 5.604, p =0.0016
WT: M=15.78, SD=9.036, n=17
Sim1-Cre: M=19.25, SD=14.54, n=20
BACHD: M=8.133, SD=6.055, n=17
BACHD/Sim1-Cre: M=9.214, SD=7.484, n=23

Tukey's multiple comparisons test:
WT vs. Sim1-Cre: p=0.7138
WT vs. BACHD: p= 0.1183
WT vs. BACHD/Sim1-Cre: p=0.1704
Sim1-Cre vs. BACHD: p=0.0058
Sim1-Cre vs. BACHD/Sim1-Cre: p=0.0076
BACHD vs. BACHD/Sim1-Cre: p=0.9862

E. Females number of open arms entries in EPM at 2 months.

One-way ANOVA, F (3, 62) = 1.003, p = 0.3977
WT: M=11.33, SD=4.63, n=21
Sim1-Cre: M=14.36, SD=7.229, n=11
BACHD: M=11.75, SD=4.171, n=16
BACHD/Sim1-Cre: M=10.94, SD=6.15, n=18

Tukey's multiple comparisons test:
WT vs. Sim1-Cre: p=0.4506
WT vs. BACHD: p=0.9957
WT vs. BACHD/Sim1-Cre: p=0.9961
Sim1-Cre vs. BACHD: p=0.6167
Sim1-Cre vs. BACHD/Sim1-Cre: p=0.3678
BACHD vs. BACHD/Sim1-Cre: p=0.9734

E. Males number of open arms entries in EPM at 2 months.

Kruskal-Wallis test: p= 0.0829
WT: M=11.91, SD=5.954, n=23
Sim1-Cre: M=16.56, SD=13.48, n=18
BACHD: M=8.538, SD=6.359, n=13
BACHD/Sim1-Cre: M=12, SD=7.502, n=29

Dunn's multiple comparisons test:
WT vs. Sim1-Cre: p> 0.9999
WT vs. BACHD: p=0.539
WT vs. BACHD/Sim1-Cre: p> 0.9999
Sim1-Cre vs. BACHD: p=0.0618
Sim1-Cre vs. BACHD/Sim1-Cre: p=0.9094

BACHD vs. BACHD/Sim1-Cre: p=0.7878

F. Females number of open arms entries in EPM at 8 months.

Kruskal-Wallis test: p= 0.5694
WT: M=9.333, SD=4.27, n=18
Sim1-Cre: M=14.2, SD=13.89, n=15
BACHD: M=10.24, SD=10.59, n=17
BACHD/Sim1-Cre: M=7.727, SD=5.742, n=22

Dunn's multiple comparisons test:
WT vs. Sim1-Cre: p> 0.9999
WT vs. BACHD: p> 0.9999
WT vs. BACHD/Sim1-Cre: p> 0.9999
Sim1-Cre vs. BACHD: p> 0.9999
Sim1-Cre vs. BACHD/Sim1-Cre: p> 0.9999
BACHD vs. BACHD/Sim1-Cre: p> 0.9999

F. Males number of open arms entries in EPM at 8 months.

Kruskal-Wallis test: p= 0.2105
WT: M=11.76, SD=8.772, n=17
Sim1-Cre: M=13.45, SD=9.099, n=20
BACHD: M=9.588, SD=10.93, n=17
BACHD/Sim1-Cre: M=8.391, SD=5.711, n=23

Dunn's multiple comparisons test:
WT vs. Sim1-Cre: p> 0.9999
WT vs. BACHD: p> 0.9999
WT vs. BACHD/Sim1-Cre: p> 0.9999
Sim1-Cre vs. BACHD: p=0.4641
Sim1-Cre vs. BACHD/Sim1-Cre: p=0.5189
BACHD vs. BACHD/Sim1-Cre: p> 0.9999

Figure 4. Assessment of the hypothalamic-pituitary-gonadal axis in male mice at 8 months.

A. Stereological quantification of GnRH positive cells in AHA

One-way ANOVA, F (3, 19) = 4.616, p =0.0137
WT: M=211.2, SD=75.23, n=5
Sim1-Cre: M=225.6, SD=49.77, n=5
BACHD: M=111.4, SD=35.13, n=7
BACHD/Sim1-Cre: M=150, SD=76.18, n=6

Tukey's multiple comparisons test:
WT vs. Sim1-Cre: p=0.9811

WT vs. BACHD: p=0.0484
WT vs. BACHD/Sim1-Cre: p=0.3621
Sim1-Cre vs. BACHD: p=0.0207
Sim1-Cre vs. BACHD/Sim1-Cre: p=0.1978
BACHD vs. BACHD/Sim1-Cre: p=0.6638

A. Stereological quantification of GnRH positive cells in rPOA

One-way ANOVA, F (3, 19) = 2.802, p =0.0677
WT: M=288, SD=55.64, n=5
Sim1-Cre: M=340.8, SD=46.16, n=5
BACHD: M=241.7, SD=47.21, n=7
BACHD/Sim1-Cre: M=308, SD=85.08, n=6

Tukey's multiple comparisons test:
WT vs. Sim1-Cre: p=0.5318
WT vs. BACHD: p=0.5753
WT vs. BACHD/Sim1-Cre: p=0.9475
Sim1-Cre vs. BACHD: p=0.0535
Sim1-Cre vs. BACHD/Sim1-Cre: p=0.8104
BACHD vs. BACHD/Sim1-Cre: p=0.239

A. Stereological quantification of GnRH positive cells in MS

Kruskal-Wallis test: p=0.772
WT: M=285.6, SD=81.12, n=5
Sim1-Cre: M=278.4, SD=52.58, n=5
BACHD: M=267.4, SD=69.87, n=7
BACHD/Sim1-Cre: M=244, SD=92.23, n=6

Dunn's multiple comparisons test:
WT vs. Sim1-Cre: p> 0.9999
WT vs. BACHD: p> 0.9999
WT vs. BACHD/Sim1-Cre: p> 0.9999
Sim1-Cre vs. BACHD: p> 0.9999
Sim1-Cre vs. BACHD/Sim1-Cre: p> 0.9999
BACHD vs. BACHD/Sim1-Cre: p> 0.9999

C. Serum testosterone level measurement

Kruskal-Wallis test: p=0.1469
WT: M=1.178, SD=2.681, n=9
Sim1-Cre: M=2.337, SD=4.683, n=9
BACHD: M=1.077, SD=2.433, n=10
BACHD/Sim1-Cre: M=1.11, SD=2.266, n=11

Dunn's multiple comparisons test:

WT vs. Sim1-Cre: p> 0.9999
WT vs. BACHD: p> 0.9999
WT vs. BACHD/Sim1-Cre: p=0.1254
Sim1-Cre vs. BACHD: p> 0.9999
Sim1-Cre vs. BACHD/Sim1-Cre: p> 0.9999
BACHD vs. BACHD/Sim1-Cre: p> 0.9999

D. Testis weight measurement

Kruskal-Wallis test: p=0.0139
WT: M=0.2478, SD=0.02172, n=15
Sim1-Cre: M=0.2477, SD=0.009956, n=8
BACHD: M=0.2664, SD=0.02087, n=8
BACHD/Sim1-Cre: M=0.2428, SD=0.01504, n=15

Dunn's multiple comparisons test:
WT vs. Sim1-Cre: p> 0.9999
WT vs. BACHD: p=0.0467
WT vs. BACHD/Sim1-Cre: p> 0.9999
Sim1-Cre vs. BACHD: p=0.1196
Sim1-Cre vs. BACHD/Sim1-Cre: p> 0.9999
BACHD vs. BACHD/Sim1-Cre: p=0.0106

Supplementary Figure 1: The number of oxytocin and vasopressin positive neurons in the PVN.

A. Females oxytocin positive cells in the PVN

Kruskal-Wallis test: p=0.8077
WT: M=1368, SD=146.6, n=4
Sim1-Cre: M=1392, SD=162, n=4
BACHD: M=1305, SD=62.45, n=4
BACHD/Sim1-Cre: M=1335, SD=121.2, n=4

Dunn's multiple comparisons test:
WT vs. Sim1-Cre: p> 0.9999
WT vs. BACHD: p> 0.9999
WT vs. BACHD/Sim1-Cre: p> 0.9999
Sim1-Cre vs. BACHD: p> 0.9999
Sim1-Cre vs. BACHD/Sim1-Cre: p> 0.9999
BACHD vs. BACHD/Sim1-Cre: p> 0.9999

A. Males oxytocin positive cells in the PVN

Kruskal-Wallis test: p=0.3185
WT: M=1437, SD=172.8, n=4
Sim1-Cre: M=1325, SD=122.9, n=4
BACHD: M=1235, SD=115.6, n=6
BACHD/Sim1-Cre: M=1367, SD=246.4, n=7

Dunn's multiple comparisons test:
WT vs. Sim1-Cre: p> 0.9999
WT vs. BACHD: p=0.461
WT vs. BACHD/Sim1-Cre: p> 0.9999
Sim1-Cre vs. BACHD: p> 0.9999
Sim1-Cre vs. BACHD/Sim1-Cre: p> 0.9999
BACHD vs. BACHD/Sim1-Cre: p> 0.9999

B. Females vasopressin positive cells in the PVN

Kruskal-Wallis test: p=0.0440
WT: M=668.1, SD=38.41, n=4
Sim1-Cre: M=806, SD=81.39, n=4
BACHD: M=645.5, SD=81.23, n=4
BACHD/Sim1-Cre: M=684.6, SD=88.25, n=4

Dunn's multiple comparisons test:
WT vs. Sim1-Cre: p=0.0934
WT vs. BACHD: p=0.9731
WT vs. BACHD/Sim1-Cre: p=0.989
Sim1-Cre vs. BACHD: p=0.0453
Sim1-Cre vs. BACHD/Sim1-Cre: p=0.155
BACHD vs. BACHD/Sim1-Cre: p=0.8807

B. Males vasopressin positive cells in the PVN

Kruskal-Wallis test: p=0.8273
WT: M=702.6, SD=121.7, n=4
Sim1-Cre: M=696.6, SD=63.36, n=4
BACHD: M=659.5, SD=55.61, n=6
BACHD/Sim1-Cre: M=694, SD=101.2, n=7

Dunn's multiple comparisons test:
WT vs. Sim1-Cre: p> 0.9999
WT vs. BACHD: p> 0.9999
WT vs. BACHD/Sim1-Cre: p> 0.9999
Sim1-Cre vs. BACHD: p> 0.9999
Sim1-Cre vs. BACHD/Sim1-Cre: p> 0.9999
BACHD vs. BACHD/Sim1-Cre: p> 0.9999

Supplementary Table 1: Hypothalamic gene expression analysis in male mice at 8 months.

Brn 2: One-way ANOVA, F (3, 30) = 0.9572, p =0.4256
WT: M=0.9989, SD=0.09048, n=9
Sim1-Cre: M=1.098, SD=0.13, n=6
BACHD: M=1.133, SD=0.2446, n=9

BACHD/Sim1-Cre: M=1.091, SD=0.1797, n=10

Tukey's multiple comparisons test:
WT vs. Sim1-Cre: p=0.7799
WT vs. BACHD: p=0.5155
WT vs. BACHD/Sim1-Cre: p=0.7799
Sim1-Cre vs. BACHD: p=0.9372
Sim1-Cre vs. BACHD/Sim1-Cre: p=0.9372
BACHD vs. BACHD/Sim1-Cre: p=0.9372

Sim1: Kruskal-Wallis test: p=0.9065
WT: M=1, SD=0.1156, n=9
Sim1-Cre: M=1.0385, SD=0.1804, n=6
BACHD: M=1.0976, SD=0.3506, n=9
BACHD/Sim1-Cre: M=1.1028, SD=0.2478, n=10

Dunn's multiple comparisons test
WT vs. Sim1-Cre: p> 0.9999
WT vs. BACHD: p> 0.9999
WT vs. BACHD/Sim1-Cre: p> 0.9999
Sim1-Cre vs. BACHD: p> 0.9999
Sim1-Cre vs. BACHD/Sim1-Cre: p> 0.9999
BACHD vs. BACHD/Sim1-Cre: p> 0.9999

Oxy: One-way ANOVA, F (3, 30) = 0.5381, p =0.6598
WT: M=1, SD=0.1098, n=9
Sim1-Cre: M=0.9367, SD=0.2229, n=6
BACHD: M=0.8733, SD=0.3733, n=9
BACHD/Sim1-Cre: M=0.999, SD=0.2086, n=10

Tukey's multiple comparisons test:
WT vs. Sim1-Cre: p=0.9814
WT vs. BACHD: p=0.8603
WT vs. BACHD/Sim1-Cre: p=0.9931
Sim1-Cre vs. BACHD: p=0.9814
Sim1-Cre vs. BACHD/Sim1-Cre: p=0.9814
BACHD vs. BACHD/Sim1-Cre: p=0.8603

Avp: Kruskal-Wallis test: p=0.1923
WT: M=1, SD=0.6461, n=9
Sim1-Cre: M=0.7733, SD=0.3983, n=6
BACHD: M=0.5378, SD=0.1995, n=9
BACHD/Sim1-Cre: M=0.677, SD=0.2993, n=10

Dunn's multiple comparisons test:

WT vs. Sim1-Cre: p> 0.9999
WT vs. BACHD: p=0.2366
WT vs. BACHD/Sim1-Cre: p=0.5856
Sim1-Cre vs. BACHD: p> 0.9999
Sim1-Cre vs. BACHD/Sim1-Cre: p> 0.9999
BACHD vs. BACHD/Sim1-Cre: p> 0.9999

SST: Kruskal-Wallis test: p=0.0512
WT: M=1, SD=0.3597, n=9
Sim1-Cre: M=0.8533, SD=0.2982, n=6
BACHD: M=0.7356, SD=0.1153, n=9
BACHD/Sim1-Cre: M=0.712, SD=0.1377, n=10

Dunn's multiple comparisons test:
WT vs. Sim1-Cre: p> 0.9999
WT vs. BACHD: p=0.1763
WT vs. BACHD/Sim1-Cre: p=0.0583
Sim1-Cre vs. BACHD: p> 0.9999
Sim1-Cre vs. BACHD/Sim1-Cre: p> 0.9999
BACHD vs. BACHD/Sim1-Cre: p> 0.9999

GHRH: Kruskal-Wallis test: p=0.0747
WT: M=1, SD=0.2831, n=9
Sim1-Cre: M=0.7567, SD=0.4071, n=6
BACHD: M=0.7778, SD=0.1021, n=9
BACHD/Sim1-Cre: M=0.742, SD=0.1623, n=10

Dunn's multiple comparisons test:
WT vs. Sim1-Cre: p=0.0841
WT vs. BACHD: p=0.6134
WT vs. BACHD/Sim1-Cre: p=0.3277
Sim1-Cre vs. BACHD: p> 0.9999
Sim1-Cre vs. BACHD/Sim1-Cre: p> 0.9999
BACHD vs. BACHD/Sim1-Cre: p> 0.9999
